# Platelet-rich plasma for the treatment of a pressure wound: a case report and review of the literature

**DOI:** 10.3389/fbioe.2026.1843937

**Published:** 2026-06-11

**Authors:** Yawen Guo, Sihui Jia, Zuzhou Huang, Jiao Liu, Zhen Zhao, Zheng Liu, Haiyan Wang

**Affiliations:** The Affiliated Hospital of Qingdao University, Qingdao, China

**Keywords:** case report, platelet, platelet-rich plasma, pressure wound, wound healing

## Abstract

**Introduction:**

Prolonged bed rest leads to pressure wounds with skin infections and difficulties in long-term wound healing. Platelet-rich plasma (PRP) contains high concentrations of platelets and is used to promote chronic wound healing.

**Objective:**

In this patient, PRP was used several times to promote the healing of a chronic pressure wound.

**Case Report:**

The patient presented with a decreased level of consciousness. Due to prolonged bed rest, a pressure wound and skin infection developed. The pressure wound was cleansed daily with iodophor, filled with alginate, and covered with a foam dressing for approximately 5 months; however, the wound did not heal. After assessment of the patient’s physical condition, autologous PRP was used in the treatment. A total of 150 mL of peripheral blood was collected from the patient for the preparation of autologous PRP. After debridement, autologous PRP and an activator (thrombin and 10% calcium chloride) were mixed in a 10:1 volume ratio and sprayed onto the surface of the pressure wound, and was given a sterile oiled gauze and gauze coverage with foam dressing for protection. After eight PRP treatments, the pressure ulcer healed within 3 months.

**Conclusion:**

The patient’s outcome suggests that PRP may be a promising adjunctive therapy for patients with nonhealing pressure ulcers.

## Introduction

Pressure wounds, also known as decubitus ulcers and pressure sores, are seen most often in high-risk populations such as older people and those with physical impairments. They develop as a result of prolonged pressure on the skin tissue, leading to tissue damage, rupture, and necrosis (Reddy et al., 2006; Boyko et al., 2018; Mervis and Phillips, 2019). The European Pressure Ulcer Advisory Panel (NPUAP) classifies pressure wounds into stages 1–4, in addition to non-stageable pressure injuries and deep tissue pressure injuries (Edsberg et al., 2016). The bony prominences of the sacrum and buttocks are the most common sites of pressure wounds (Leblebici et al., 2007). Localized skin infections are common complications of pressure wounds. If not treated promptly, the infection penetrates deep into the muscle layer, bones, and joints, leading to serious consequences such as muscle infection and necrosis, joint infection, osteomyelitis, sepsis (Jaul, 2010). Pressure wounds aggravate the physical suffering of patients, leading to increased mortality and decreased quality of life, and imposing severe burdens and economic losses on individuals, their families, and society. Presently, the treatment of pressure wounds includes pressure relief, nutrition, management of chronic conditions, adequate drainage, debridement, and wound care. If the condition is severe, with ulceration, exudate, or necrosis, it is usually treated with regular dressing changes after routine debridement (Boyko et al., 2018). In addition, the effectiveness of dressings in the prevention of pressure wounds remains controversial (Clark et al., 2014). With an aging population, there is an urgent clinical need for effective treatment of pressure wounds.

Platelet-rich plasma therapy promotes tissue repair and regeneration by collecting and concentrating autologous platelets and is administered via injections or topical applications. The main component of PRP is a high concentration of platelets; therefore, it contains many alpha granules and dense granules. When platelets in PRP are activated by activators such as thrombin and calcium chloride, these concentrated granules release vital growth factors and cytokines: platelet-derived growth factor, transforming growth factor-β, vascular endothelial growth factor, epidermal growth factor, and others. Research has shown that PRP therapy is an important tool for promoting the healing of chronic wounds and minimizing scar formation (Guo et al., 2017; Hesseler and Shyam, 2019). PRP has also been used in orthopedics, dentistry, and ophthalmology. Although PRP has been moderately studied in chronic wounds, to date, reports on elderly patients with long-term nonhealing pressure ulcers remain limited. This case highlights potential benefit in this population (Sell et al., 2011). In this case, PRP may be a promising adjunctive therapy.

## Case report

**FIGURE 1:**
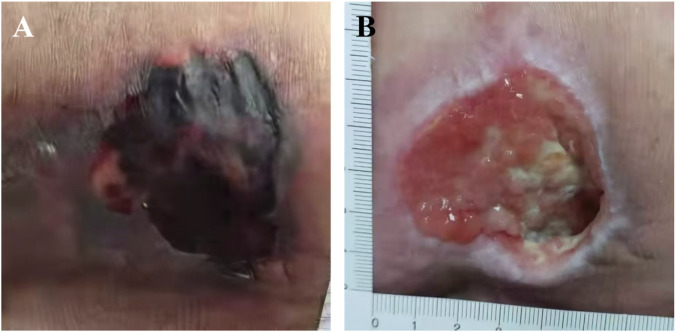
**(A)** Initial state of pressure ulcer. **(B)** Status of pressure ulcer after 2 months.

An 88-year-old female patient presented on 19 July 2021, with a decreased level of consciousness. The patient’s medical history was complex, with a history of tuberculosis 50 years prior, cured with oral medication; chronic bronchitis for more than 10 years, with symptomatic treatment for acute symptoms; cerebral atrophy for 9 years; coronary artery disease for 7 years; and Alzheimer disease for more than 10 years, which was treated with medication but had been discontinued. Because the patient was chronically bedridden, the admission examination showed that there was a 14 cm × 14 cm unstageable pressure ulcer combined with a skin infection on the left buttock (Figure 1A). Due to the patient’s overall poor health, conservative management was chosen. Upon admission, the nursing staff sterilized the wound and protected it with foam dressings twice daily. In addition, the patient was administered an albumin infusion to promote healing of the pressure wound. On 29 September 2021, the wound was found to have more exudate with 80% red tissue and 20% yellow tissue visible (Figure 1B); 0.5% iodophor was used to disinfect the wound, which was then rinsed with saline, and alginate and a foam dressing was placed to protect the wound twice a day. Treatment was performed until 21 December 2021, when the examination showed that the pressure ulcer was stage 3, measuring 5 cm × 4 cm × 1 cm. Compared to admission, the patient’s wound was slightly improved, but not healed. At baseline, the patient’s albumin level was 30.10 g/L, C-reactive protein level was 30.41 mg/L, procalcitonin level was 0.860 ng/mL, and leukocyte count was 18.12 × 10^9^/L. Microbiological culture of the wound yielded *Escherichia coli* and *Staphylococcus aureus*, while sputum culture detected *Candida albicans*. The coagulation indices remained within the normal range. Anti-infective treatments administered during hospitalization included linezolid and amikacin sulfate injection.

**FIGURE 2:**
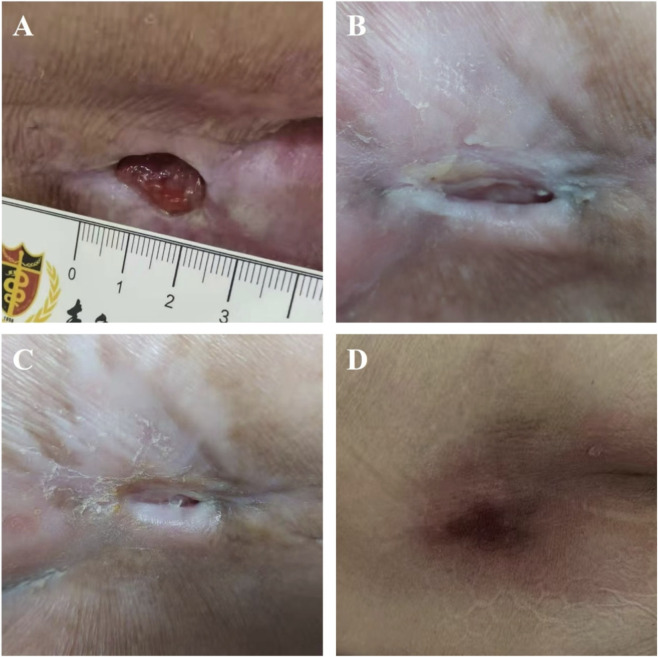
**(A)** After two PRP treatments, the wound began to shrink. **(B)** After the third PRP treatment, the wound was reduced in size to 2 cm × 1 cm × 0.5 cm. **(C)** At the eighth PRP treatment, the ulcer shrinking to 1 cm × 1 cm × 0.1 cm. **(D)** The wound is completely healed.

Considering that the pressure wound had not healed for more than 5 months, autologous PRP treatment was recommended. Before initiating PRP therapy, the clinician provided the patient with a thorough explanation of the purpose and risks of therapy. At the end of the discussion, the patient and legal guardian voluntarily agreed to participate and signed an informed consent form. On 25 December 2021, a 150 mL autologous venous blood sample was obtained and transferred to a pre-sterilized triple blood bag containing 3.8% sodium citrate anticoagulant. Plasma was centrifuged (1,300 rpm, 12 min) at 22 °C ± 2 °C to separate plasma and red blood cells (Hitachi CR7, Japan). The remainder was centrifuged again (3,200 rpm, 15 min) to separate the PRP and platelet-free plasma. The obtained PRP was left at 22 °C ± 2 °C for 1–2 h before use. Blood was stored in sterile, sealed blood storage bags. The patient’s baseline platelet count was 123 × 10^9^/L, and the PRP concentration was 698 × 10^9^/L (Myriad BC-2600; China). After debridement, 5 mL of autologous PRP and an activator (thrombin and 10% calcium chloride) were mixed in a 10:1 volume ratio and sprayed onto the surface of the pressure wound, and was given a sterile oiled gauze and gauze coverage with foam dressing for protection. A second PRP treatment was performed on the same day, and the patient did not complain of any discomfort during the PRP treatment. Two days later, the patient’s pressure ulcer were significantly reduced in size (Figure 2A), followed by a third PRP treatment on 7 January 2022. 20 January 2022, the wound was reduced in size to 2 cm × 1 cm × 0.5 cm (Figure 2B), with approximately 80% fresh granulation tissue visible in the wound bed. Subsequently, the fourth to eighth PRP treatments were carried out on January 21 and 22, February 15 and 22, and 22 March 2022. At the final treatment (Table 1), the left buttock pressure ulcer decreased significantly, shrinking to 1 cm × 1 cm × 0.1 cm (Figure 2C). On 26 March 2022, the wound healed completely without scarring (Figure 2D), both the patient and patient’s legal guardian expressed satisfaction with the treatment outcome. The decision to complete eight PRP sessions was based on the patient’s wound response, wound-bed condition, and the treating team’s clinical judgment, rather than on a standardized protocol.

**TABLE 1:** Timeline of PRP treatment sessions.

Date	Session
2021-12-25	1st and 2nd PRP
2022-01-07	3rd PRP
2022-01-21	4th PRP
2022-01-22	5th PRP
2022-02-15	6th PRP
2022-02-22	7th PRP
2022-03-22	8th PRP

## Discussion

Pressure wounds are common, usually combined with a variety of diseases. They are difficult, lengthy, and costly to treat, and lack a uniform treatment method. Pressure wounds are primarily treated conservatively. Current management of pressure ulcers is multimodal and includes pressure redistribution, optimization of nutritional status, infection control, wound-bed preparation, debridement, exudate management, appropriate dressings, and surgical reconstruction when indicated. The choice of topical medication affects the length of the healing period and is the most researched treatment (Gould et al., 2024). Surgical options such as adjacent flap transfer are also available if surgery is indicated. In the present case, the ulcer remained unhealed after approximately 5 months of conventional wound care, and autologous PRP was therefore considered as a potential adjunctive treatment.

In recent years, a considerable number of studies have reported that PRP has a restorative effect on chronic wounds (Ramos-Torrecillas et al., 2013; Wang et al., 2024). The ratio of the concentration of various growth factors contained in PRP is close to the normal ratio *in vivo*, which is more advantageous than single growth factor treatment in promoting wound healing (Everts et al., 2020). PRP may have contributed to granulation tissue formation and wound-bed repair through growth factor release and fibrin scaffold formation (Chicharro-Alcántara et al., 2018). Following platelet activation, alpha and dense granules are secreted. Among them, adenosine diphosphate (ADP) establishes a positive feedback loop by activating the P2Y12 receptor, which continuously amplifies the activation signal (Entsie et al., 2023). At the same time, the αIIbβ3 integrin undergoes a conformational change, exposing the ligand-binding site (Huang et al., 2019). Fibrinogen binds to activated integrins and mediates platelet cross-linking, building a three-dimensional network scaffold that supports cell migration and neovascularization, and ultimately promotes wound repair. To date, there is no standardized procedure for the preparation of PRP, which means that the optimal effective concentration, timing, and dosage of PRP and the method of preservation are unknown, and that differences in platelet concentration, activation method, treatment interval, and application volume may affect clinical outcomes. Further clinical trials are needed to confirm the feasibility of allogeneic donation of PRP *in lieu* of therapy (Fadadu et al., 2019). However, autologous PRP treatment has no risk of disease transmission and has fewer adverse reactions; its effect is obvious and can significantly shorten the treatment period (Suthar et al., 2017). Therefore, PRP was considered as a potential adjunctive treatment option in this patient. However, although this case suggests that topical autologous PRP may be a feasible adjunctive option for selected patients with difficult-to-heal pressure ulcers, controlled studies are required to determine its independent efficacy and optimal protocol.

## Limitations

In this report, the patient responded to PRP treatment with a significant reduction in the volume of the pressure ulcer and an increased rate of healing, however, some issues still need to be considered. First, the single-case design limits generalizability, as this report describes a single patient, improvement observed in this patient may also be influenced by nutritional support, infection management, and natural healing progression. Thus, the results should be interpreted as supportive clinical observations rather than definitive evidence of efficacy. Second, while fully utilizing the excellent regenerative effects of PRP, it is important to consider its possible adverse effects. Older patients or patients with underlying diseases such as hypertension and diabetes may have a long history of medication use before receiving PRP treatment, and certain antiplatelet drugs may have potential effects on the function and status of platelets. Etulain et al. (2018) showed that PRP-mediated angiogenesis was eliminated after pretreatment of PRP with the antiplatelet drug acetylsalicylic acid, suggesting that antiplatelet medications may negatively affect PRP efficacy. Because the patient presented with decreased consciousness and advanced Alzheimer disease, reliable patient-reported pain and quality-of-life assessments were not feasible. Additionally, Wound healing was assessed by measurements and qualitative observation, no standardized scoring system was used, which may introduce measurement bias.

## Conclusion

In this patient, PRP treatment was chosen with informed consent and caution owing to the patient’s advanced age and limited surgical tolerance. PRP may be a promising adjunctive therapy for nonhealing pressure ulcers, however, controlled studies and continued follow-up are needed to confirm efficacy. To date, only two studies on the use of PRP for the treatment of pressure ulcers has been reported (Sell et al., 2011; Ramos-Torrecillas et al., 2013); however, further experimental studies are required to test for the efficacy of PRP treatment of pressure wounds. The outcome in this patient suggests that the future use of PRP may help develop individualized treatment plans for patients with non-healing pressure ulcers.

## Data Availability

The original contributions presented in the study are included in the article/supplementary material, further inquiries can be directed to the corresponding author.
